# Effects of Free and Conjugated Methionine on Growth, Meat Quality, Mineral Profile, and Shell Strength in Garden Snails (*Cornu aspersum*)

**DOI:** 10.3390/ani15192922

**Published:** 2025-10-08

**Authors:** Anna Rygało-Galewska, Klara Piotrowska, Magdalena Matusiewicz, Damian Bień, Monika Łukasiewicz-Mierzejewska, Zbigniew Skibko, Andrzej Borusiewicz, Tomasz Niemiec

**Affiliations:** 1 Department of Animal Breeding and Nutrition, Institute of Animal Sciences, Warsaw University of Life Sciences, Ciszewskiego 8 Street, 02-786 Warsaw, Poland; klara_piotrowska@sggw.edu.pl (K.P.); damian_bien@sggw.edu.pl (D.B.); monika_lukasiewicz@sggw.edu.pl (M.Ł.-M.); tomasz_niemiec@sggw.edu.pl (T.N.); 2 Institute of Technology and Life Sciences—National Research Institute, Falenty, 3 Hrabska Avenue, 05-090 Raszyn, Poland; 3 Department of Nanobiotechnology, Institute of Biology, Warsaw University of Life Sciences, 166 Nowoursynowska St., 02-787 Warsaw, Poland; magdalena_matusiewicz@sggw.edu.pl; 4 Faculty of Electrical Engineering, Bialystok University of Technology, Wiejska 45, 15-351 Bialystok, Poland; z.skibko@pb.edu.pl; 5 Department of Agronomy, Modern Technologies and Informatics, International University of Applied Sciences in Lomza, 18-402 Lomza, Poland; andrzej.borusiewicz@mans.edu.pl

**Keywords:** edible snails, *Cornu aspersum*, snail nutrition, methionine addition

## Abstract

**Simple Summary:**

This study evaluated the effects of methionine (Met) and its conjugated form (Met-Met) on the growth performance, carcass composition, mineral profile, and shell quality of *Cornu aspersum* snails under controlled laboratory conditions. Two experiments tested different Met inclusion levels (0.3, 0.6, and 1.4 g/kg feed) and compared free Met, Met-Met, and their mixture (1.4 g/kg feed). The highest Met dose (1.4 g/kg) significantly improved body weight, shell mass, and shell crushing force, while also increasing carcass Met content and enhancing shell mineralisation, particularly calcium. Among treatments, Met-Met supplementation yielded the greatest carcass-to-body weight ratio, the highest proportion of mature individuals, and indications of improved copper absorption and storage. These results demonstrate that methionine supplementation can enhance productivity, product quality, and commercial value in snail farming by improving shell resistance and carcass nutritional properties, especially in Met-Met form.

**Abstract:**

The present study examined the impact of adding methionine (Met) and its conjugated form (Met-Met) on *Cornu aspersum* snails. The primary focus was on the animals’ growth performance, the chemical composition of their carcass (whole body without the shell), the mineral profile, and the mechanical properties of their shells. In two experiments conducted under controlled laboratory conditions, diets supplemented with varying levels of Met addition (0.3, 0.6, 1.4 g/kg feed) were used, and the effects of free methionine, Met-Met and their mixture (1.4 g/kg feed) were compared. The study incorporated measurements of body weight, shell width, and mortality of snails. Analyses encompassing protein, fat, sulphur amino acids, glutathione levels, oxidative stress indices (DPPH, TAC, TBARS), and macro- and micronutrient content of carcass and shells were conducted. The findings demonstrated that adding 1.4 g Met/kg feed significantly enhanced the shells’ weight gain (+56% vs. Control), shell weight (+56%) and crushing force (+135%). Furthermore, an increase in the Met content of the carcass was observed (+18%), along with elevated carcass Ca (+28%) and P (+30%) and higher shell Ca (+12%) and Zn (+87%), alongside reduced carcass Fe (−38%) and Cu (−19%). In Experiment II, the Met-Met group exhibited the highest carcass weight (+16% vs. Control), the greatest carcass-to-body weight ratio, and the highest proportion of mature individuals (+27%). Moreover, Met-Met supplementation improved Cu absorption and retention in the carcass (+19%). Also, the results suggest that the conjugated form of methionine may improve Cu absorption and storage in the carcass (+19%). The study’s findings indicate that methionine addition, especially in Met-Met form, can substantially impact the efficiency of *C. aspersum* farming, enhancing both the productivity outcomes and the quality of the product. That is particularly important in increasing the shell’s mechanical resistance and the carcass’s nutritional value.

## 1. Introduction

Researchers are exploring alternative animal protein sources to satisfy the rising demand associated with the growth of the human population. Edible snails represent a promising option due to many factors, including fast growth cycles, low cost of animal husbandry, cost-effectiveness, and environmentally friendly production methods [1,2,3]. Snail meat and caviar are relatively low in calories and are becoming increasingly appreciated for their culinary versatility. In several European seaside countries (such as Italy, France, and Greece), snails are used in traditional cuisine, with high and consistent demand for production [4,5]. Snail meat constitutes a valuable source of protein (comprising 59.53–67.42% of dry matter), unsaturated fatty acids, essential amino acids, vitamins and minerals [6].

A significant challenge in the farming of snails is the assurance of sufficient shell strength and integrity. The fragility of the shells can complicate processing steps, such as mechanical cleaning, sorting, and long-distance transport, which can result in financial losses and a reduction in the product’s suitability for export. That is particularly relevant for garden snails (*Cornu aspersum*), traditionally served in their shells. The shells account for approximately one-third of a snail’s body weight and perform several essential functions, including protecting internal organs, preventing dehydration, shielding from cold temperatures, and defending against predators and pathogens [7,8]. A snail’s shell is composed of approximately 95–99% calcium carbonate [9].

Methionine, a sulfur-containing essential amino acid, offers a range of physiological benefits. As the initial amino acid in protein synthesis, methionine is vital for cellular growth and maintenance [10,11]. Furthermore, it functions as a precursor for S-adenosylmethionine (SAM), a universal methyl donor in the methylation of DNA, RNA, and proteins, influencing gene expression, cellular signalling, and epigenetic regulation [12,13]. This methylation capacity impacts various biological functions, from gene activation and cellular differentiation to metabolic control, thereby underscoring methionine’s foundational importance in development and health [14]. Furthermore, methionine plays a role in antioxidant defence systems by synthesising glutathione. This potent antioxidant helps maintain cellular redox balance and protects cells from oxidative damage, which is particularly crucial under stress conditions [15,16].

The availability of methionine in feed-grade forms, such as DL- and L-methionine, allows for the creation of tailored dietary formulations that ensure optimal protein intake [17]. The supplementation of methionine has been demonstrated to confer benefits across various livestock species. In poultry, methionine supplementation has enhanced feed conversion ratios, protein synthesis, and growth performance [18]. In swine, methionine supplementation increases daily weight gain and improves carcass quality, with increased lean muscle mass and reduced fat deposition [19]. Also, it was proven that methionine enhances the bioavailability of copper, zinc, and cobalt in growing lambs [20].

The value of methionine extends to its role as a precursor for other essential metabolites, including creatine, glutathione, and phosphatidylcholine. These metabolites offer notable benefits for animals regarding muscular, hepatic, and neurological health [21]. For example, creatine has been demonstrated to support muscle development and endurance [22]. While methionine is essential, it necessitates cautious administration, as excessive supplementation can result in toxicity issues, such as oxidative stress and metabolic imbalances. Furthermore, excessive methionine intake may also disrupt the homeostasis of other amino acids, potentially impairing growth and health [23,24]. However, current research indicates that methionine requirements may be elevated during periods of stress, suggesting that augmented supplementation levels could confer additional benefits to immune resilience and metabolic efficiency [23].

The role of methionine supplementation in the nutrition of invertebrates has been the subject of study, among other species, in the context of shrimp nutrition [25] in which, among others, the conjugated form of methionine (Met-Met) was used [26,27]. These studies showed that the administration of this amino acid exerts a beneficial effect on animal growth. At the same time, some research has examined the role of methionine in the nutrition of marine molluscs, including species such as scallops *Mytilus edulis* and clams *Rangia cuneata* [28], flat oysters *Ostrea angasi*, blue mussels *Mytilus edulis planulatus*, and Sydney cockles *Anadara trapezia* [29]. In abalone *Haliotis Discus Hannai*, optimal dietary methionine levels (0.97–1.19%) have been demonstrated to enhance growth, protein synthesis, immunity, and heat stress resistance [30]. Similarly, in Chinese mitten crabs, a methionine-enriched diet has been shown to promote growth, muscle protein deposition, immunity, and antioxidant capacity [31]. It was proven by Kintsu et al. [32] that the shells of pearl oysters contain methionine-rich proteins that may be important for scaffold formation (initial biomineral formation). Therefore, the presence of methionine in molluscs’ diets may be crucial for accumulating shell elements. The study by Hunt [33] showed that methionine is present in the matrix of *Helix pomatia* snails, alongside other amino acids.

Despite the well-documented importance of methionine supplementation in livestock and aquatic species, its role in the nutrition of terrestrial molluscs such as *C. aspersum* remains largely unexplored. This study aimed to address this gap by providing novel insights into how methionine and its conjugated form, Met-Met, affect growth performance, carcass quality, mineral composition, and shell strength in edible snails, as it was proven to have higher bioavailability in shrimps [26,34]. The goal was to optimise the growth and condition of snails through improved dietary strategies, as this topic remains poorly explored despite the well-known critical functions of amino acids in other species.

## 2. Materials and Methods

### 2.1. Experimental Design

Two independent feeding trials were conducted to evaluate the effects of dietary methionine supplementation in *Cornu aspersum* snails on fattening results (body weight, shell width, mortality), shell parameters and quality (shell indices and crushing force), and the nutritional quality of the snail carcass (proximate composition, amino acid profile, oxidative status, and mineral content).

Snails were maintained at the Warsaw University of Life Sciences animal facility, with a mechanical-gravity ventilation system. Both experiments were performed under identical housing and environmental conditions (controlled using a hygrometer and a thermometer, 22 ± 1 °C, 60 ± 10% relative humidity, 12 h light/12 h dark cycle; humidity was maintained by daily manual misting in the boxes). The mobile racks holding the containers were relocated biweekly, and the containers on each shelf were rotated weekly.

Hatchlings aged 2–3 days (mean body weight 0.02 g, mean shell width 4.0 mm) were obtained from a commercial breeder and randomly allocated to experimental containers (10 L plastic boxes with sterilised soil substrate, 35 snails per container, three replicate containers per treatment, giving 105 individuals per group). The mobile racks holding the containers were relocated biweekly, and the containers on each shelf were rotated weekly.

Animals were fed *ad libitum* as a flour mixture of constant composition (Table 1), exchanged twice a week to maintain freshness. The feed formulation was based on prior research and INRA standards [35,36]. Calcium carbonate was chosen as the Ca source due to its purest and most effective form [37,38]. The premix contained only vitamins and minerals, with no added amino acids. Methionine was added as a free amino acid or a conjugated amino acid (Met-Met; AQUAVI MetMet; EVONIK). As fodder yeast (*Saccharomyces cerevisiae*) was used in the feed as a protein source, it was a part of a standardised diet, and its level remained constant (4%). Methionine was the only dietary variable, added either in free form or as a dipeptide (Met-Met).

In Experiment I, four diets were compared: a control diet without additional methionine and three diets supplemented with 0.3, 0.6, or 1.4 g free Met/kg feed. This experiment aimed to establish the optimal inclusion level of free methionine in snail nutrition. The level of added methionine was increased by 10%, 20% and 40% relative to the baseline level of methionine in the feed. Similar levels of methionine in feed were previously used in chicken [39,40] and shrimps [26,41,42].

**Figure 1:**
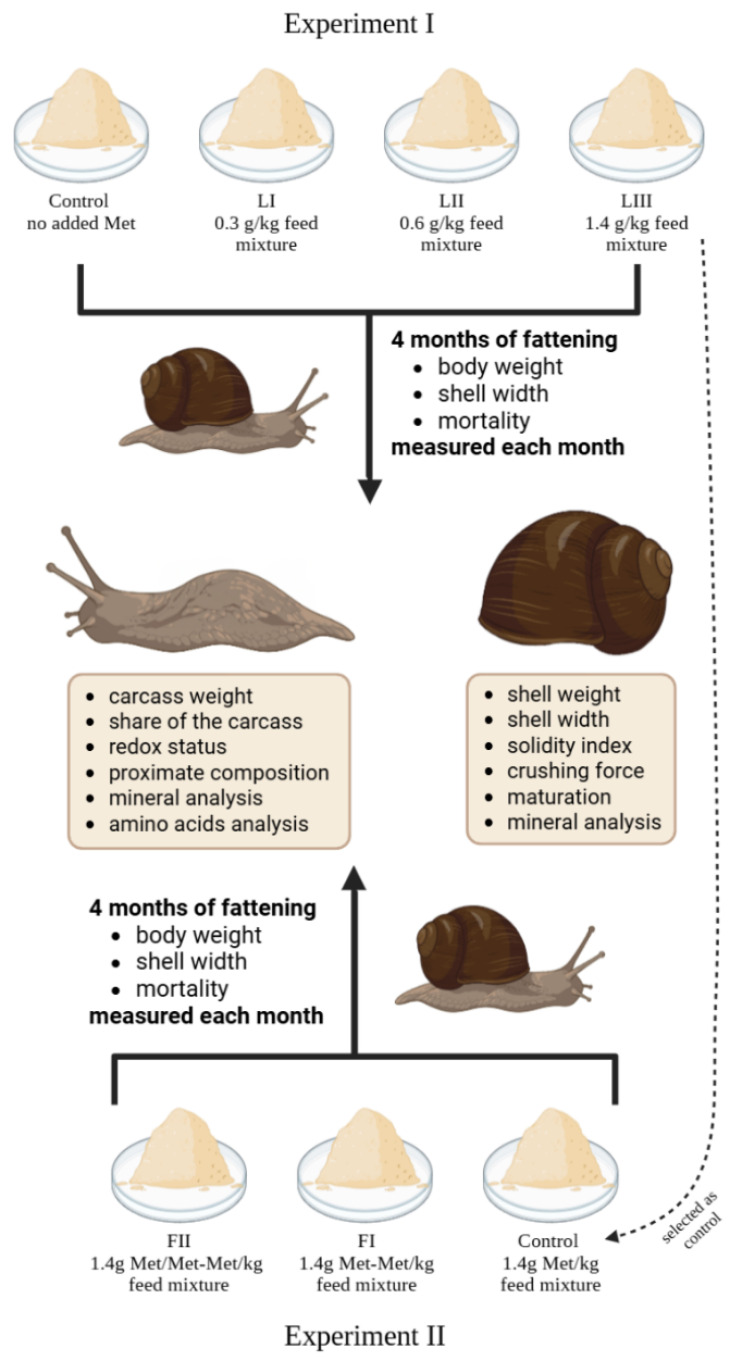
Schematic representation of the design of Experiments I and II: including group categorisation based on methionine content and form, snail measurement methodology, and analyses at termination of the study.

In Experiment II, all diets contained the methionine level identified as most effective in Experiment I (1.4 g/kg feed), but differed in the form of supplementation: the Control group received only free Met, group FI received only Met-Met, and group FII received a mixture of 50% free Met and 50% Met-Met. This experiment aimed to compare the effects of methionine forms at an identical total supplementation level (Figure 1).

In both trials, body weight, shell width (measured with a calliper—AOS ABSOLUTE Digimatic Standard, Mitutoyo, Japan), and mortality were recorded monthly in a random subsample of 50 individuals per group. At the end of the experimental period, 50 randomly chosen snails from each treatment were fasted for 24 h, euthanised by freezing (−80 °C), and analysed for carcass (proximate composition according to AOAC methods, amino acid profile by an AAA-500 analyser, oxidative status by DPPH, TAC, TBARS and glutathione assays) and shell characteristics (mineral composition by ICP-OES, shell morphology indices, and mechanical strength by Zwicki-Line crushing force test). The entire body of a snail without the shell was considered a carcass, reflecting its culinary use.

### 2.2. Experimental and Analytical Procedures

At the beginning of the laboratory analyses, the snails were removed from their shells, and the weights of the carcasses and shells were recorded. The proportion of carcass weight to total body weight (including shell mass) was calculated for a randomly selected sample of fifty snails from both Experiment I and Experiment II. Subsequently, further analyses were conducted to determine the chemical and amino acid composition, the redox status of the snail carcass, and the mineral composition of both the carcass and the shells.

The chemical composition of the snail carcasses was determined following the methods set forth by the AOAC (Association of Official Analytical Chemists) [43]: The dry matter content was determined by drying the samples at 105 °C until a constant weight was achieved. Crude ash was determined through incineration at 550 °C for six hours. The crude protein content was determined using the micro-Kjeldahl technique (Kjeltec System 1026 Distilling Unit, Foss Tecator, Sweden), while the crude fat content was assessed through extraction with petroleum ether using the Soxhlet method.

Thiobarbituric acid reactive substances (TBARSs) were quantified as malondialdehyde (MDA) equivalents using 1,1,3,3-tetraethoxypropane (TEP) as a standard for the preparation of the calibration curve. The methodology outlined by Uchiyama and Mihara [44] was employed. The snail carcass tissues were homogenised in a 1% potassium chloride solution and subjected to centrifugation at 2000 rpm for 15 min at 4 °C. The resulting filtrate was combined with 1% phosphoric acid, 1% potassium chloride, 2% butyl hydroxyanisole (BHA), and 0.4% thiobarbituric acid (TBA). Following thorough mixing, the samples were sealed and placed in a water bath at 100 °C for 60 min. Once the mixture had cooled, 4 mL of butanol was added, and the contents were shaken for 2 min. The absorbance of the compounds dissolved in the butanol phase was determined at a wavelength of 532 nm. The results were expressed in nmol/mL, derived from the standard curve against TEP.

OxiSelect™ Total Antioxidant Capacity (TAC) Assay Kit (STA-360, Cell Biolabs, INC.) enables the quantification of overall antioxidant levels in various sample types through a single electron transfer (SET)-based method. This assay relies on the ability of antioxidants to reduce copper ions from their Cu^2+^ (copper II) state to Cu^+^ (copper I). Freeze-dried snail carcass tissues were weighed and homogenised after adding methanol to achieve a 2000 µg/L concentration. The resulting homogenate was centrifuged at 10,000× *g* for 10 min at 4 °C. 20 µL of the supernatant was combined with 180 µL of reaction buffer in a 96-well microplate. The initial absorbance at 490 nm was measured using a plate reader (Tecan, Männedorf, Switzerland). Subsequently, 50 µL of the Copper Ion Reagent was added to each well. The plate was incubated for 5 min on an orbital shaker, followed by adding 50 µL of Stop Solution to terminate the reaction. Absorbance was measured again at 490 nm. Net absorbance was calculated by subtracting the initial absorbance values from the final readings for each sample. Results for the Experimental Groups were then expressed as a percentage relative to the Control Group.

The antioxidant potential of snail tissue was evaluated using the DPPH free radical scavenging assay. Freeze-dried snail samples were pulverised and dissolved in methanol to achieve a final 2000 µg/L concentration. The solution was vortexed at 2000 rpm for 5 min and filtered using a Whatman filter. To perform the assay, 10 µL of the sample was combined with 290 µL of 0.1 M DPPH solution. After a 20-min incubation in the dark, absorbance was measured at 570 nm using a plate reader (Tecan, Männedorf, Switzerland). Antioxidant activity was expressed as the percentage of DPPH radical inhibition.

Glutathione is quantified by determining non-protein-SH Groups in deproteinised samples using the Ellman method. It involves the reduction of DTNB (5,5′-dithiobis (2-nitrobenzoic acid)) by thiols and the formation of coloured 2-nitro-5-mercaptobenzoic acid, with maximum absorbance at 412 nm [45,46]. Freeze-dried and ground bodies of *C. aspersum* snails (50 mg) were homogenised in cold 0.1 M phosphate buffer pH 7.4 (1 mL), using a bead homogeniser with a cold adapter (TissueLyser LT, Qiagen, Hilden, Germany; 50 L/s, 15 min) and centrifuged (10,000× *g*, 15 min, 4 °C). The obtained extracts (500 μL) were deproteinised by mixing them with 50% trichloroacetic acid (TCA; 26.32 μL) and centrifugation (10,000× *g*, 15 min, 4 °C). The supernatants were frozen in liquid nitrogen and stored (at −80 °C). The next day, extracts (25 μL) were placed in a 96-well plate, mixed with 0.2 M phosphate buffer pH 8.0 (200 μL) and then with 6 mM DTNB (25 μL). The absorbance was measured using an Infinite M200 microplate reader (Tecan, Männedorf, Switzerland). GSH in 2.5% TCA (0–75 nmol/mL) was used to construct the standard curve. Number of replicates (n) = 3; each replicate included 8 individuals.

The amino acid content was analysed using an AAA-500 chromatographic amino acid analyser (INGOS, Prague, Czech Republic) following modified protocols [47]. Hydrolysis was employed for sulfur-containing amino acids. Freeze-dried and ground samples of snail bodies were treated with a 2.5 mL mixture of formic acid and hydrogen peroxide (9:1) and incubated at 4 °C for 16 h. The reaction was halted by adding 0.5 mL of concentrated hydrochloric acid. Subsequently, acid hydrolysis was performed using 40 mL of 6 M HCl at 125 °C for 23 h. The cooled hydrolysate was filtered through Whatman 3 paper into 100 mL volumetric flasks and topped with demineralised water. Hydrochloric acid was evaporated at 35 mbar pressure in a 50 °C water bath. The dry residues were dissolved in a citric buffer with a pH of 2.6. For the analysis, buffers with pH 2.6 and 3.0 were utilised, the column temperature was set to 58 °C, and the reactor temperature to 121 °C [48]. Calculations were made by referencing external standards using the Clarity 10 software.

The mineral content of the snail carcasses and shells was analysed by inductively coupled plasma optical emission spectrometry (ICP-OES). The samples were mineralised in a chamber furnace at 450 °C for 12 h. Subsequently, acid digestion was conducted using a specified volume of 37% hydrochloric acid (AnalaR NORMAPUR^®^, VWR Chemicals, Radnor, PA, USA) on a hotplate. The digested material was filtered through blotting paper, and the resulting solution was diluted to 50 mL with distilled water. The total concentrations of calcium (Ca), copper (Cu), iron (Fe), magnesium (Mg), phosphorus (P), zinc (Zn), sodium (Na), chromium (Cr), cobalt (Co), cadmium (Cd) and lead (Pb) were determined using ICP-OES equipment (Perkin Elmer Avio 200; Waltham, MA, USA).

To ascertain the maturity of the snails, it was necessary to determine whether a shell lip was present, which indicates the maturity of the snail [8]. Shell dimensions were measured to calculate the shell shape index, which is defined as the shell width-to-height ratio and the solidity index. That method was adapted from the assessment of poultry eggs [49,50] and previously applied to snail shells by Ligaszewski et al. [51] employs the following formula: The formula for calculating shell weight is as follows: shell weight × (height/width) × 100.

The crushing force of the shells was measured by using a Zwick 1120 machine (Z 5.0 Zwicki-Line, Ulm, Germany) equipped with a Warner–Bratzler blade set with a force of 0.2 N. Static tests were conducted at a constant speed of 5 mm/min. The compressive strength was measured based on the resistance that shells provided.

### 2.3. Statistical Analysis

Data were evaluated using one-way analysis of variance (ANOVA) in Statistica 13.3 (TIBCO Software Inc., Palo Alto, CA, USA), with significance defined at *p* < 0.05. Post-hoc comparisons were conducted using the LSD test, and repeated measures ANOVA was applied where appropriate.

The statistical model applied was Yij = μ + αi  +  eij, where Yij is the observed value, μ is the overall mean, αi denotes the effect of the experimental diet (i.e., methionine level in Experiment I or form in Experiment II), and eij is random error.

Repeated measures ANOVA was performed using PS IMAGO PRO 9.0 software for body weight, shell width, and mortality. Significant interaction effects were further examined with pairwise comparisons applying the Bonferroni correction. All variables satisfied the sphericity assumption, and statistical significance was accepted at *p* < 0.05.

## 3. Results

**Figure 2:**
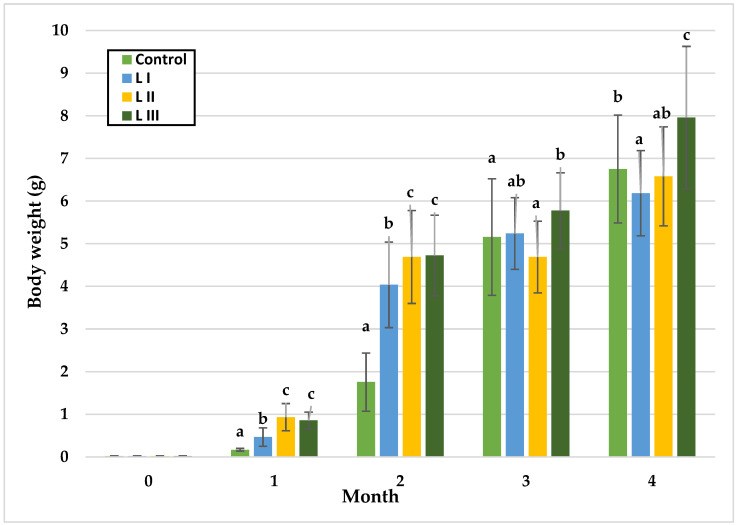
Monthly body weight (g) of snails in Experiment I. Data are shown as mean ± SD (n = 50). Control: no Met; LI, LII, LIII—diets with 0.3, 0.6, and 1.4 g/kg Met level, respectively. The means indicated with different superscripts (a, b, c) are significantly different (*p* < 0.05).

**Figure 3:**
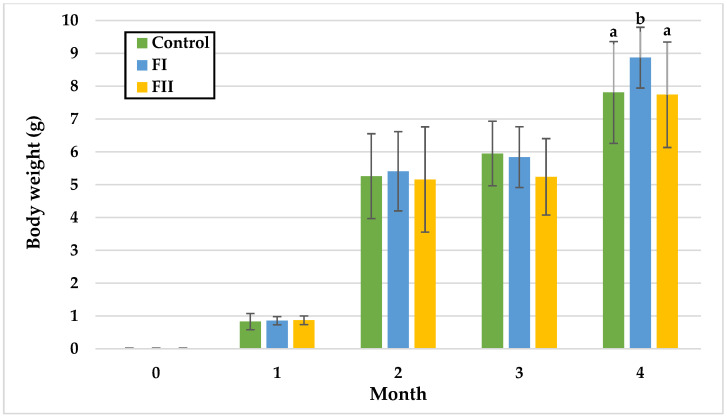
Monthly body weight (g) of snails in Experiment II. Data are shown as mean ± SD (n = 50). Control: 1.4 g/kg Met; FI: Met-Met; FII: 50% Met + 50% Met-Met. The means indicated with different superscripts (a, b) are significantly different (*p* < 0.05).

**Figure 4:**
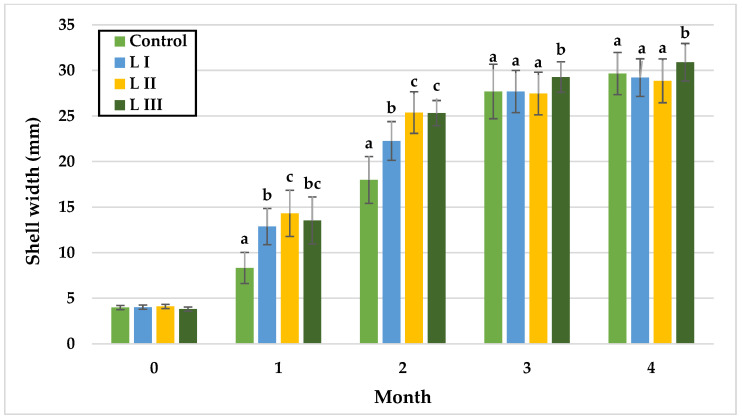
Monthly shell width (mm) of snails in Experiment I. Data are shown as mean ± SD (n = 50). Control: no Met; LI, LII, LIII—diets with 0.3, 0.6, and 1.4 g/kg Met level, respectively. The means indicated with different superscripts (a, b, c) are significantly different (*p* < 0.05).

**Figure 5:**
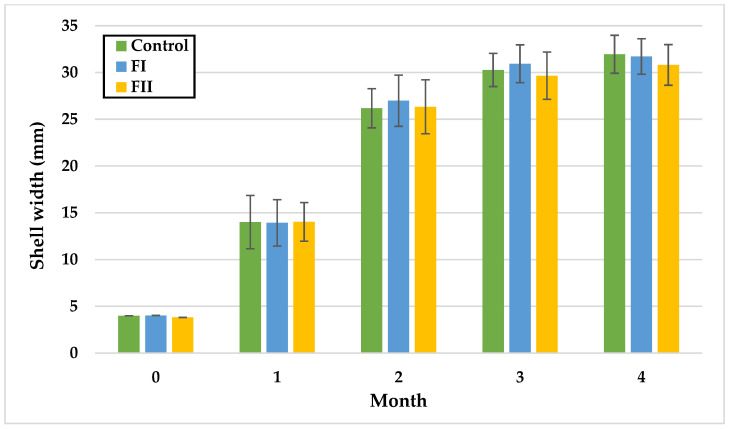
Monthly shell width (mm) of snails in Experiment II. Data are shown as mean ± SD (n = 50). Control: 1.4 g/kg Met; FI: Met-Met; FII: 50% Met + 50% Met-Met.

**Figure 6:**
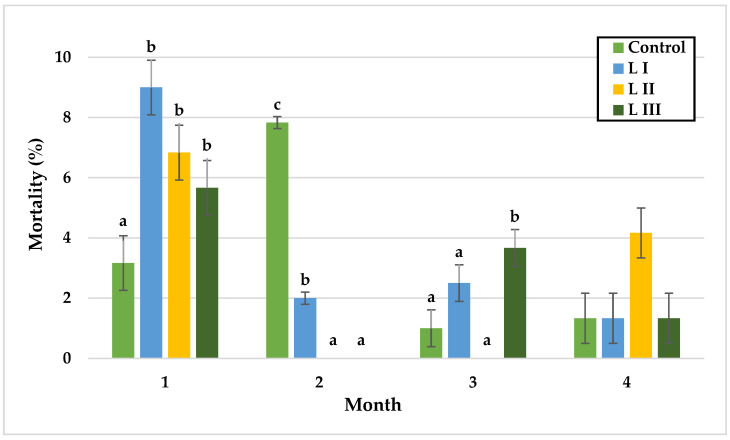
Monthly mortality (%) of snails in Experiment I. Data are shown as mean ± SD (n = 3). Control: no Met; LI, LII, LIII—diets with 0.3, 0.6, and 1.4 g/kg Met level, respectively. The means indicated with different superscripts (a, b, c) are significantly different (*p* < 0.05).

**Figure 7:**
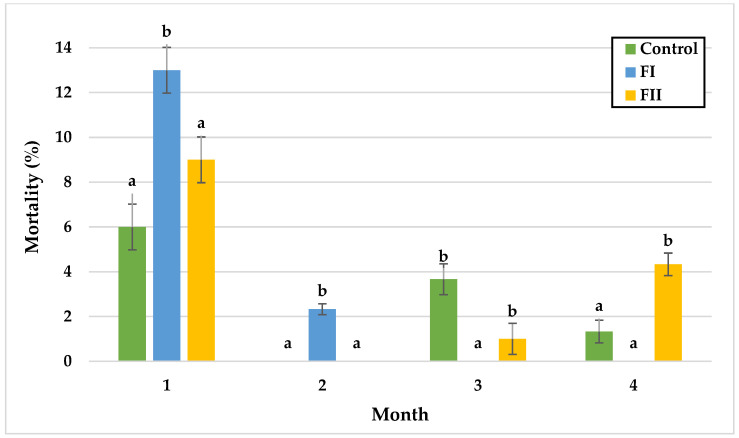
Monthly mortality (%) of snails in Experiment II. Data are shown as mean ± SD (n = 3). Control: 1.4 g/kg Met; FI: Met-Met; FII: 50% Met + 50% Met-Met. The means indicated with different superscripts (a, b) are significantly different (*p* < 0.05).

The growth of snails occurs in three phases: during the first month, there is slow body growth coupled with rapid internal organ development; in the second and third month, body weight increases rapidly. After 13 weeks of age, body weight stabilises, and growth begins to slow down [52]. The data collected from monthly measurements in both Experiment I and Experiment II are illustrated in Figure 2, Figure 3, Figure 4, Figure 5, Figure 6 and Figure 7.

In Experiment I, significant body weight (BW) differences among groups emerged over time. During the first and second months, BW did not differ between the LII and LIII groups, while the Group FI and the Control differed significantly. At the end of month one, L II exhibited the highest mean BW (0.93 g) and the control the lowest (0.17 g). By month two, the highest BW was observed in the LIII Group (4.71 g), with the Control remaining lowest (1.76 g). In month three, significant differences were detected between the Control and LIII Group, and between the LII and LIII groups, with mean BW reaching 4.69 g (LII), and 5.78 g (LIII). In the final month, LIII Group attained the highest mean BW (7.96 g), significantly exceeding the Control (6.75 g), while LI Group recorded the lowest BW.

In Experiment II, BW remained statistically similar among groups during the first three months. Significant differences appeared only in the fourth month, with the FI Group showing the highest mean BW (8.87 g), compared to the Control (7.81 g) and FII Group (7.74 g).

In Experiment I, during the first month, no differences were observed between LI and LIII or between LII and LIII, with the lowest mean SW in the control group (8.33 mm) and the highest in L II (14.33 mm). In month two, SW did not differ significantly between LII and LIII, while the control remained lowest (17.99 mm). By month three, significant differences were detected between the control and LIII, LI and LIII, and LII and LIII, with LIII reaching the highest mean SW (29.27 mm) and the other groups showing similar values (control: 27.69 mm; L I: 27.68 mm; L II: 27.47 mm). In the fourth month, LIII exhibited significantly greater SW (30.90 mm) compared with LI (29.21 mm) and LII (28.86 mm).

In Experiment II, no statistically significant differences in SW were observed over time. At the end of the fourth month, the highest mean SW was recorded in the control group (31.95 mm), and the lowest in FII (30.81 mm).

In Experiment I, significant differences in snail mortality were observed primarily during the first and third months. In the first month, mortality was lowest in the control group (3.2%) and highest in LI (9%). During the second month, mortality peaked in the control group (7.8%) without significant differences among groups. By the third month, L II exhibited the lowest mortality (0%), whereas LIII reached the highest rate (3.7%). In the final month, mortality rates did not differ significantly, with values of 1.3% in the control, LI, and LIII groups, and 4.2% in LII.

In Experiment II, mortality remained similar between the control and FII groups during the first two months, with the lowest rate in the control group (6.0%) and the highest in FII (9.0%) during month one. In the third month, mortality was significantly lower in FI (0%) and FII (1%) compared with the control (3.7%). In the final month, significant differences were observed between the control and FII, and between FI and FII, with the highest mortality in FII (4.3%) and the lowest in FI (0%), followed by the control (1.3%).

In Experiment I, dietary treatments significantly affected multiple growth and shell parameters (Table 2). Carcass weight was highest in Group LIII (6.62 g), exceeding values observed in the Control and other experimental groups. Shell weight also increased significantly in LIII (1.20 g), differing from all other groups. Conversely, the proportion of carcass in total body weight was lowest in the LIII Group (84.72%) relative to the Control and LI–LII groups. The shell shape index was reduced in LI Group (1.23) and LII Group (1.14) compared with the Control (1.42), while the solidity index reached its maximum in the LIII Group (14.13) and minimum in the LI Group (10.63). Shell crushing force was markedly elevated in the LIII Group (48.05 N), significantly exceeding that of the other groups.

In Experiment II, carcass weight was significantly higher in Group FI (7.65 g) than in the Control (6.57 g) and FII Group (6.62 g). The proportion of carcass in total body weight also increased in both experimental groups, reaching 86.20% in the FI Group and 85.66% in the FII Group, compared with 84.53% in the Control. Additionally, the shell shape index was elevated in the FII Group (1.29) relative to the Control (1.16) and the FI Group (1.19). In contrast, no significant differences were observed for shell weight, solidity index, shell crushing force, or the proportion of mature individuals.

Table 3 presents the proximate composition and amino acid content of snail carcasses. In Experiment I, crude protein content was significantly higher in groups LI (71.18%) and LII (69.83%) than in the Control and LIII groups. Ether extract content was reduced in LII (1.75%) relative to the other groups, with the highest value observed in LI (2.75%). Methionine concentration increased in LIII (11.03 mg/g DM), while the control group showed the lowest level (9.30 mg/g DM). No significant differences were found for crude ash or cysteine content.

In Experiment II, ether extract content was significantly lower in both experimental groups (1.71–1.86%) than in the control (2.40%). Methionine concentration was also affected, with higher values in the FI Group (11.92 mg/g DM) and the Control (11.05 mg/g DM). No significant differences were noted for crude protein, crude ash, or cysteine.

In Experiment I, pronounced differences in mineral composition were observed (Table 4). Carcass calcium increased from 1.16% in the Control to 1.64% in the LI Group, accompanied by a parallel rise in shell calcium, with the highest concentration recorded in the LIII Group (38.74%). Carcass phosphorus was elevated in the LI Group (14,236 mg/kg) and LII Group (13,623 mg/kg) compared with the Control (10,942 mg/kg), while shell phosphorus also increased, reaching 655 mg/kg in the LI Group versus 363 mg/kg in the Control. Carcass zinc content rose in the LI Group (88.52 mg/kg) and LII Group (82.43 mg/kg), though values in the LIII Group (68.64 mg/kg) returned to control levels; shell zinc displayed a similar pattern, remaining higher in all experimental groups (3.84–4.86 mg/kg) than in the Control (2.60 mg/kg). By contrast, carcass iron decreased progressively from 110.90 mg/kg in the Control to 68.39 mg/kg in L III, accompanied by a parallel decline in shell iron, lowest in the LIII Group (13.77 mg/kg). Carcass sodium content declined from 8142 mg/kg in the Control to 6454 mg/kg in the LIII Group. Carcass copper decreased from 63.64 mg/kg in the Control to 51.77 mg/kg in the LIII Group, while shell copper slightly reduced across all treatments. Cadmium levels were highest in the Control (0.96 mg/kg) and decreased significantly in all experimental groups, reaching 0.52 mg/kg in the LII Group. Chromium and cobalt were detected sporadically, while lead was absent in all samples.

In Experiment II, dietary effects were more selective. Shell calcium was reduced in both experimental groups (36.11–36.95%) relative to the Control (38.74%). Shell iron content increased markedly in the FII Group (23.18 mg/kg) compared with the Control (13.76 mg/kg) and FI Group (12.13 mg/kg). Shell sodium rose in the FII Group (947 mg/kg) compared with the Control (792 mg/kg). Carcass copper concentrations increased in both experimental groups, from 51.97 mg/kg in the Control to 61.87 mg/kg in the FI Group and 57.46 mg/kg in the FII Group. In contrast, shell copper declined in the FII Group (6.77 mg/kg). Trace amounts of chromium were detected in shells, with higher levels in FII Group (0.91 mg/kg) compared to minimal concentrations in the Control (0.16 mg/kg). No significant differences were found in carcass calcium (1.39–1.50%), carcass and shell phosphorus, carcass iron (65–69 mg/kg), carcass sodium (5918–6452 mg/kg), carcass zinc (68–72 mg/kg), shell zinc (4.28–5.39 mg/kg), or carcass cadmium (0.57–0.61 mg/kg). Cobalt and lead were not detected.

**Figure 8:**
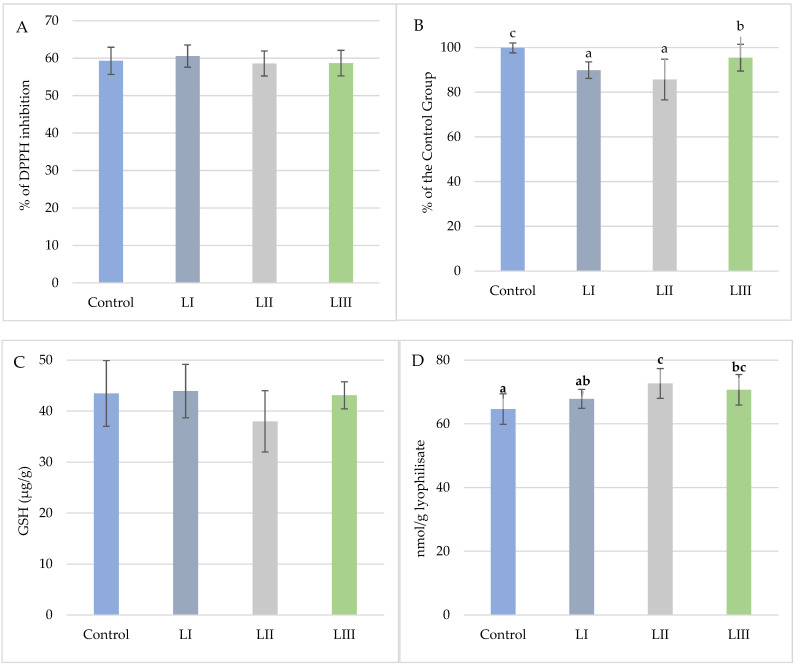
(**A**). Mean values (±SD, n = 15) % DPPH inhibition of snail carcasses in Experiment I. (**B**). Mean values (±SD, n = 15) of snail carcasses’ total oxidative potential (TAC) in Experiment I. (**C**). Mean content (±SD, n = 3) of glutathione (μg/g) in snail carcasses in Experiment I. (**D**). Mean values (±SD, n = 15) of TBARS of snail carcasses in Experiment I. No-Met Control diet, L I, L II, and L III diet with Met dose, respectively: 0.3, 0.6, and 1.4 g/kg feed. The means indicated with different superscripts (a, b, c) are significantly different (*p* < 0.05).

In Experiment I, dietary treatments significantly affected TAC and TBARS values (Figure 8). TAC was highest in the LIII Group (95.44) and lowest in the LII Group (85.67), representing a 14.1% decrease compared with the Control. As measured by TBARS, lipid peroxidation was elevated in the LII Group (72.72 nmol/g) relative to the Control (64.65 nmol/g). DPPH inhibition and glutathione content did not differ significantly among groups.

**Figure 9:**
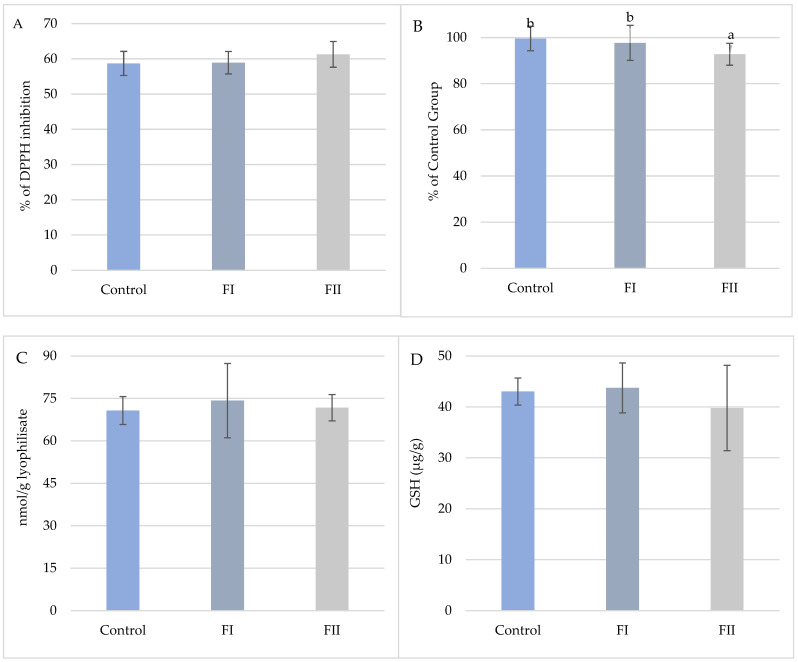
(**A**). Mean values (±SD, n = 15) % DPPH inhibition of snail carcasses in Experiment II. (**B**). Mean values (±SD, n = 15) of total oxidative potential (TAC) of snail carcasses in Experiment II. (**C**). Mean values (±SD, n = 15) of TBARS of snail carcasses in Experiment II. (**D**). Mean content (±SD, n = 3) of glutathione (μg/g) in snail carcasses in Experiment II. Met Control diet, FI—Met-Met, FII—50% Met and 50% Met-Met. The means indicated with different superscripts (a, b) are significantly different (*p* < 0.05).

In Experiment II, only TAC was significantly affected by dietary treatment (Figure 9). TAC was highest in the FI Group (97.70%) and decreased in the FII Group (92.78%), representing a 6.8% reduction relative to the Control. No significant differences were observed for DPPH inhibition, glutathione content, or TBARS values.

## 4. Discussion

### 4.1. Growth Rates

The findings corroborate the three-phase course of snail growth, wherein, during the initial month, a gradual escalation in body mass is observed, concomitant with pronounced development of internal organs. At this stage, the absence of substantial disparities between groups in body weight suggests that methionine supplementation did not yet influence somatic growth, likely due to the priority of internal organ development. However, as growth progressed, marked differences emerged. In Experiment I, significant body weight gains (*p* < 0.05) were observed in the second and third months, with the highest values recorded in Group LIII receiving 1.4 g/kg methionine. This trend persisted until the conclusion of the study, indicating that methionine may play a pivotal role in promoting development during the dynamic growth phase, as also reported for common carp (0.67% Met in feed) [53], broiler chicken (0.37% Met in feed) [40], and white shrimp (1.01% Met in feed) [34]. Comparable results were found in abalones (1.19% Met in feed) [30], sea cucumbers (up to 0.57% Met in feed) [54], and silver catfish (up to 1.27–1.37% Met in feed) [55], where growth increased proportionally with methionine levels until a threshold was reached, beyond which toxic effects became apparent. Although the levels of methionine applied in this study were limited, it cannot be excluded that higher concentrations could provoke similar adverse effects in snails. A comparable stimulatory effect of methionine was observed for shell width, with Group LIII again showing the greatest values throughout the experiment (*p* < 0.05).

In contrast, Experiment II revealed no statistically significant differences (*p* < 0.05) in body weight among groups during the first three months, indicating that the form of amino acid (Met vs. Met-Met) did not substantially affect growth during the early intensive phase. Significant differences (*p* < 0.05) appeared only in the fourth month, when Group FI (Met-Met supplementation) reached the highest mean body weight (8.87 g). These results suggest that the dipeptide form possesses greater bioavailability than free methionine, likely due to active absorption via intestinal peptide transporters and more efficient utilisation in metabolic pathways. Considering the standardised environmental and rearing conditions, the disparities in snail growth observed in this study can be ascribed chiefly to the provision of methionine in the diet and its formulation.

Similar benefits of Met-Met supplementation were reported in Nile tilapia [56] (0.15–0.21% Met-Met in feed) and white shrimp (0.12% and 0.17% Met-Met in feed) [26,57]. In mammals, di- and tripeptide forms of methionine are known to be 15–76% more effective in protein synthesis than equivalent doses of free methionine, for example, in mammary gland tissues, where Met-Met improved milk protein production and lactation efficiency [58]. Thus, the growth differences observed here are primarily ascribed to dietary methionine provision and its formulation. Unlike Experiment I, no significant variation in shell width was detected, suggesting that methionine form did not affect shell development in this trial.

Mortality analysis across both experiments provides additional insight into the impact of methionine supplementation on snail condition. In Experiment I, the Control Group snails showed the lowest mortality (*p* < 0.05) during the first month. In contrast, higher rates were observed in methionine-supplemented groups, suggesting that exceeding 0.3 g/kg may increase early mortality risk. In subsequent months, however, mortality consistently declined in the supplemented groups, implying a longer-term protective effect of methionine. That aligns with evidence of methionine’s anti-ageing properties in various species [59] and its reported role in reducing mortality in laying hens (0.38–0.44% Met in feed) [39]. In Experiment II, Group FI (Met-Met) showed zero mortality in the third and fourth months, in contrast to Control and FII, which exhibited higher values (*p* < 0.05). This outcome suggests that the dipeptide form improves resilience, consistent with findings in shrimp where Met-Met (0.18–0.24% and 0.94% in feed) enhanced survival [34,42].

### 4.2. Carcass Characteristics

The results concerning carcass traits highlight the importance of methionine supplementation and its formulation in shaping nutrient utilisation and tissue development. In Experiment I, the highest carcass weight was observed in Group LIII, corresponding to an 8.4% increase over the Control (*p* < 0.05), which indicates that free methionine supplementation effectively stimulates soft tissue growth [60]. This effect may be explained by enhanced methionine availability supporting structural and enzymatic protein synthesis [14] and reduced energy losses through mitigation of oxidative stress [61]. Comparable findings were reported in Chinese mitten crabs, where supplementation (up to 1.25% Met in feed) improved growth performance and antioxidant status [31]. In Experiment II, significant differences were observed, with Group FI (Met-Met supplementation) reaching the highest carcass weight (7.65 g), 16.4% higher than the Control (6.57 g; *p* < 0.0001). The superior performance of Met-Met suggests that the conjugated dipeptide form is more bioavailable than free methionine, which is consistent with studies *in Litopenaeus vannamei* shrimps (0.18–0.24% Met-Met in feed) [26]. These findings indicate that while free and dipeptide methionine enhance carcass deposition, the bioavailable Met-Met form exerts a stronger late-phase effect.

Protein and fat composition further underscore the influence of methionine form and dose. In Experiment I, the highest crude protein content was recorded in the LI Group (*p* < 0.01), reflecting efficient protein utilisation. However, in Group LIII, despite the highest carcass weight, crude protein content did not peak, suggesting that the increase in body mass also resulted from elevated deposition of carbohydrates in the form of glycogen and galactose deposition as energy reserves for wintering [62,63]. This pattern resembles responses observed in Chinese mitten crabs (up to 1.25% Met in feed) [31] and sea cucumbers (up to 0.78% Met in feed) [54], where protein content increased with supplementation before declining at higher doses. In Experiment II, concerning sulphur amino acids, the Met-Met Group exhibited a significantly elevated methionine content (*p* < 0.01), suggesting a favourable impact of Met-Met supplementation on the amino acid profile of the carcass. That trend was also observed in common carp (up to 0.58% Met-Met in feed) [64], and white shrimp (0,1% and 0.18–0.24% Met-Met in feed) [26,34]. However, given that the amount of protein in the carcass remained constant, but the amount of methionine increased, it is possible that the presence of methionine in the intercellular spaces and the cells themselves, where it was used for various metabolic processes, including methylation and synthesis of chemical compounds [65].

Fat content also varied: in Experiment I, Group LI showed the highest and LII the lowest values (*p* < 0.01), indicating shifts in lipid metabolism. The parallel effect was observed in sea cucumbers fed methionine (up to 0.57% in feed) [54]. In Experiment II, the lowest fat content (*p* < 0.01) was observed in Group FI (Met-Met), suggesting reduced lipogenesis and greater reliance on lipids as an energy source. A comparable effect was observed in shrimp (0.2–0.3% Met-Met in feed), where fat content decreased with increasing dipeptide supplementation [66].

As methionine is a precursor of SAM, it regulates methylation reactions affecting lipid metabolism and carnitine synthesis [60,67,68]. Methionine has also been demonstrated to influence energy metabolism and the storage of reserve components. In chickens, a deficiency of methionine has been shown to result in impaired transport of lipids in the form of very-low-density lipoproteins (VLDLs) from the liver, resulting in atosis and impaired growth [69]. Similarly, in fish (*Megalobrama amblycephala*), a methionine-deficient diet (0.40% of the diet) caused a significant increase in liver lipid content, with a simultaneous decrease in fat and glycogen content in the muscles [70]. In that study, methionine deficiency was demonstrated to inhibit TOR pathway activity in the liver, thereby exerting a deleterious effect on energy production and protein synthesis. Methionine restriction has also been linked to enhanced lipolysis and reduced fat accumulation [71]. An excessive methionine supply in the diet has been demonstrated to promote fat mobilisation and oxidation. In chickens, high concentrations of methionine increase expression of β-oxidation enzymes, including ACOX1 and LPL, and concurrently reduce fat accumulation in the liver [69]. In fish, a diet with a high methionine content (1.28%) has been observed to stimulate the transcription of genes involved in gluconeogenesis in the liver (pepck, G6P) and glycolysis in muscles (pfk, pk) [70]. These results indicate that an increased supply of methionine intensifies the processing of energy substrates, increasing lipid oxidation and glucose availability. Methionine influences lipid and carbohydrate metabolism by modulating glutathione biosynthesis and signalling pathways such as TOR. A deficiency of this amino acid has resulted in excessive fat accumulation in the liver and a reduction in muscle glycogen stores.

Methionine content in carcasses reflected supplementation patterns. In Experiment I, the highest concentrations were recorded in Group LIII (*p* < 0.05), contrary to shrimp studies, where methionine addition (up to 0.95% Met-Met in feed) did not alter meat content [42]. The elevated methionine concentration in snail carcasses may reflect an increased antioxidant capacity, enhancing the organism’s defence against oxidative stress. This hypothesis could be substantiated by the oxidative stress and antioxidant capacity indices, which were significant supplementary factors in evaluating carcass quality. In Experiment II, methionine content was elevated in Met-Met group (*p* < 0.01), aligning with findings in common carp (up to 0.58% Met-Met in feed) [64] and white shrimp (0.1% and 0.18–0.24% Met-Met in feed) [26,34]. Since total protein remained stable, the higher methionine levels likely indicate its storage in tissues and intercellular spaces, supporting methylation and biosynthetic processes [65].

Elemental composition also responded to methionine supplementation. In Experiment I, Group LI displayed the highest concentrations of Ca, P, and Zn (*p* < 0.01), suggesting improved bioavailability of minerals with moderate methionine addition, consistent with results in other species [72,73]. Elevated crude ash content in LI (Met addition at 0.3 g/kg feed) further supports the hypothesis of intensified mineral storage in tissues rather than shells. In contrast, snails in the Control Group exhibited higher Fe, Cu, and Na concentrations (*p* < 0.01), possibly reflecting unregulated mineral deposition. Similar reductions in Cu were reported in broiler chickens fed excess methionine (1.5%) [74], and decreased Co levels in carcass tissues were previously linked to methionine supplementation [75]. In Experiment II, significant differences were limited to higher Cu content (*p* < 0.01) in Group FI, suggesting that Met-Met may enhance Cu absorption through intestinal complex formation, similar to Cu–methionine complexes in pigs [76]. The present results provide the first evidence that Met-Met enhances Cu absorption in land snails by forming soluble complexes within the intestinal lumen, as in pigs. In this study, methionine supplementation in the free form resulted in reduced Cu concentrations in the carcass at each level of supplementation.

Antioxidant status provides additional insight into the dual effects of supplementation. In Experiment I, Group LI showed the highest glutathione levels and DPPH inhibition (*p* < 0.05), suggesting that moderate supplementation (0.3 g/kg) optimises antioxidant defence. The enhanced antioxidant status may have been instrumental in promoting both growth efficiency and meat quality by mitigating oxidative damage to proteins and lipids, as it was proven in Chinese mitten crab (higher gene expression levels of anti-lipopolysaccharide factor 1 (ALF1), prophenoloxidase (proPO), Crustin-1, cap ‘n’ collar isoform C (CncC) as well as activities of adenosine deaminase (ADA), glutamate transaminase (GPT) and aspartate aminotrafserase (GOT) up to 1.25% Met in feed) [31] and *Haliotis discus hannai* abalones (glutathione content and total antioxidative capacity in cell-free hemolymph (CFH) were elevated at 1.19% Met in feed) [30]. However, Groups LII and LIII exhibited elevated TBARS, indicating increased lipid peroxidation despite higher glutathione levels, suggesting that intensive growth may raise oxidative load beyond the neutralising capacity of the antioxidant system. High methionine doses are known to exert toxic effects in various species [77,78].

The increased body size observed in groups supplemented with methionine may be associated with an elevated metabolic rate and intensified oxidative and antioxidative processes [79,80]. In Experiment II, antioxidant indices differed mainly in TAC (*p* < 0.05), which was lowest in Group FII. Other markers, including TBARS, glutathione, and DPPH, showed no significant changes (*p* < 0.05), indicating a stable oxidative environment. Improved antioxidant abilities linked to Met-Met supplementation were observed previously in shrimps (reduced malondialdehyde (MDA) concentration, increasing ACP, LZM and T-AOC activities, upregulating gene expression level of immune and TOR signalling pathways) [57], and white leg shrimps (reduced MDA and elevated phenol oxidase content, peroxidase and TAC at 0.15% Met-Met in feed) [66].

The observed discrepancy between Experiment I and Experiment II can be attributed to variations in the oxidative challenge across trials. In Experiment I, the TBARS values were significantly higher in the control group. This finding indicates that the yeast-derived antioxidants in all diets were insufficient to prevent lipid peroxidation entirely, and that additional free methionine further reduced TBARS. In contrast, Experiment II revealed that overall TBARS levels were lower across all groups. That suggests that under these conditions, the contribution of yeast, combined with a generally lower oxidative load, was sufficient to maintain lipid stability, and supplementation with Met-Met did not further decrease lipid peroxidation. It is also noteworthy that TBARS, as an indicator of secondary lipid oxidation products, exhibits heightened sensitivity to pronounced differences instead of subtle variations in oxidative status. That may have contributed to the uniform outcomes observed in Experiment II.

The administration of the highest dose of methionine (LIII, 1.4 g/kg) resulted in the greatest increase in body growth and shell strength and increased TBARS. Accelerated protein accretion and biomineralisation enhance mitochondrial flux and ROS generation, elevating lipid peroxidation. However, the increase in TAC in LIII, combined with the unaltered DPPH scavenging activity and stable GSH, indicates that the antioxidant defence system was not depleted but increased to match the higher metabolic load. In Experiment II, Met-Met further enhanced TAC and growth performance without elevating TBARS, showing that the bioavailable dipeptide form can support anabolic processes while limiting oxidative imbalance. The findings suggest that snails are not insensitive to oxidative stress but are capable of mounting compensatory antioxidant responses adequate to their metabolic rate, thereby keeping oxidative challenges within a physiological adaptation range.

### 4.3. Characteristics of Shells

The shell of molluscs serves not only as a protective structure but also as a reservoir of minerals, offering valuable insights into the environmental and nutritional conditions under which the animal developed [81,82]. The biomineralisation process relies on the organised deposition of calcium carbonate, guided by organic macromolecules such as proteins and polysaccharides. In particular, calcium-binding proteins regulate the availability of Ca^2+^ ions, while matrix proteins provide scaffolds for mineral deposition [83]. That indicates that dietary methionine may indirectly shape shell properties by enhancing protein synthesis and facilitating mineral incorporation.

The results of both experiments demonstrate that methionine supplementation influences shell growth, strength, and elemental composition, although with differing magnitudes depending on the form and dose applied. In Experiment I, the highest recorded shell weight, solidity index (14.13 g/cm^2^ × 100; *p* < 0.01), and crushing force (48.05 N; *p* < 0.01) were observed in Group LIII, supplemented with 1.4 g/kg of free methionine. This outcome suggests that high methionine intake promoted the intensification of biomineralisation processes, possibly by stimulating the synthesis of matrix proteins such as conchiolin, which serve as a structural framework for calcium deposition [9]. The observed increase in shell strength may also be attributed to more efficient utilisation of available mineral resources and elevated activity of the cells responsible for mineral secretion within the snail’s shell. That is supported by evidence that methionine-rich insoluble proteins are involved in the biomineral formation of pearl oysters [32]. Correspondingly, the Ca content of shells was greatest in Group LIII (*p* < 0.01), likely accounting for the increased weight and robustness of shells [84,85]. The elevated Ca content of the shell, when considered in relation to the Ca content of the carcass, may indicate a prior retention of Ca utilisation for structural purposes over metabolic utilisation. It is worth noticing that Fe and Cu content (*p* < 0.01) in shells in the Met-receiving groups was lower than in the Control Group, the trend opposite to the one observed in the carcass. At the same time, Zn, Na, and P levels were significantly elevated (*p* < 0.01) compared to the Control Group. Since molluscs are known to use their shells as mineral reservoirs and detoxification sites [86], this redistribution suggests that methionine supplementation alters mineral allocation between shell and soft tissues. Methionine has been demonstrated to be involved in shell biomineralisation. As Kintsu et al. [32] explain, several shell matrix proteins identified in bivalves contain methionine-rich domains that contribute to the organic scaffold for CaCO_3_ deposition. Consequently, the enhanced availability of Ca and Zn in the presence of methionine may be directly linked to stronger shell formation through improved mineral supply and incorporation into the shell matrix. The present results in *C. aspersum* provide the first evidence that these mechanisms may also operate in land snails, as higher doses of Met increased shell strength.

Experiment II revealed no statistically significant differences (*p* > 0.05) in shell morphometry or strength between groups, aside from the shell shape index. Nevertheless, changes in mineral profiles were detected. The Ca content of the Control Group shells was the highest (*p* < 0.01), indicating the influence of free methionine form on this mineral distribution in snails. The FII Group had the highest content of Na and Fe (*p* < 0.01). The Cu content was highest (*p* < 0.05) in the Control and FI Groups. This finding aligns with the results observed in the carcass composition of snails in the FI Group. It may suggest either enhanced bioavailability of Cu or its improved retention caused by the form of methionine in the diet [76,87].

An additional aspect linking shell quality to broader physiological processes is maturation. In Experiment I, the highest proportion of mature individuals was observed in Group LIII (54% vs. 42% in the Control, trend *p* < 0.1), suggesting that methionine supplementation may accelerate reproductive development. That is consistent with its role as a precursor for cysteine, taurine, and SAM, compounds involved in hormone synthesis and reproductive signalling [88,89]. Taurine plays an essential role in reproductive processes, and its biosynthesis is closely related to the availability of methionine in the diet. Studies on female white shrimp (*Penaeus vannamei*) have demonstrated that taurine supplementation significantly affects the increase in the gonadal specific index (GSI) [90]. Furthermore, an elevated rate of gonadal maturation was observed in the supplemented groups, thereby suggesting a beneficial effect of taurine, and thus indirectly methionine, on fertility. A similar set of relationships was identified in the relevant studies on mammals. It has been demonstrated that an augmented methionine provision during the sexual cycle promotes ovarian follicle maturation and oestrogen synthesis [91].

## 5. Conclusions

The results of this study indicate that both the form and level of dietary supplementation significantly affect the growth and quality of *C. aspersum* snails, particularly regarding carcass and shell traits. Methionine supplementation increased body weight, improved mineral composition, and enhanced shell strength. Notably, 1.4 g/kg feed addition of methionine showed the best shell mechanical properties and the highest percentage of sexually mature snails. Also, it led to marked gains in body and shell weight, improvements in Ca deposition, and reduced Fe and Na in carcasses.

Comparing methionine forms, the group fed Met-Met achieved better growth and carcass yield than the mixed-form group, though not all mineral or biochemical parameters improved. The experiment results suggest that the conjugated form of methionine may enhance Cu absorption in land snails, as free methionine supplementation reduced Cu concentrations in the carcass.

Optimising methionine dose and form could boost production efficiency and product quality, with practical benefits such as higher body weight of snails (resulting in higher earnings for the breeder) and stronger shells during handling and increased market value. These findings highlight the importance of tailoring feeding strategies for snails and monitoring their physiological effects on animals.

## Figures and Tables

**Table 1:** Ingredients and determined the chemical composition of snail feed in Experiments I and II.

Ingredients (%)	Experiment I				Experiment II		
	Control	L I	L II	L III	Control	FI	FII
Corn meal	43.80	43.80	43.80	43.79	43.79	43.79	43.79
Soybean meal 460	21.00	21.00	21.00	21.00	21.00	21.00	21.00
Wheat bran	5.00	5.00	5.00	5.00	5.00	5.00	5.00
Fodder yeast#break# *Saccharomyces cerevisiae*	4.00	4.00	4.00	4.00	4.00	4.00	4.00
Rapeseed oil	2.00	2.00	2.00	2.00	2.00	2.00	2.00
Monocalcium phosphate	2.00	2.00	2.00	2.00	2.00	2.00	2.00
Calcium carbonate	20.00	20.00	20.00	20.00	20.00	20.00	20.00
Sodium Chloride	0.20	0.20	0.20	0.20	0.20	0.20	0.20
Vitamin premix	2.00	2.00	2.00	2.00	2.00	2.00	2.00
Methionine (g)	-	0.30	0.60	1.40	1.40	-	0.70
Met-Met (g)	-	-	-	-	-	1.40	0.70
**Composition (% DM)**							
Crude protein	17.88 ± 0.58	18.40 ± 0.45	18.78 ± 0.61	19.15 ± 0.73	19.20 ± 0.55	18.94 ± 0.49	19.12 ± 0.51
Ether extracts	3.01 ± 0.10	3.16 ± 0.09	3.11 ± 0.11	2.97 ± 0.12	3.06 ± 0.11	3.07 ± 0.08	2.91 ± 0.10
Crude fibre	3.33 ± 0.09	3.50 ± 0.08	3.21 ± 0.08	3.22 ± 0.09	3.60 ± 0.10	3.39 ± 0.08	3.16 ± 0.09
Crude ash	27.24 ± 0.79	28.39 ± 0.57	27.76 ± 0.67	27.61 ± 0.52	28.32 ± 0.92	27.74 ± 0.83	27.22 ± 0.71
Nitrogen-free extract	48.02 ± 1.66	46.55 ± 1.33	47.14 ± 0.89	47.05 ± 1.20	45.82 ± 0.52	46.86 ± 0.61	47.59 ± 0.98
Methionine (mg/g DM)	2.94 ± 0.10	3.22 ± 0.20	3.51 ± 0.14	4.27 ± 0.15	4.31 ± 0.18	4.42 ± 0.36	4.26 ± 0.11
Cysteine (mg/g DM)	2.60 ± 0.05	2.55 ± 0.05	2.37 ± 0.07	2.14 ± 0.03	2.12 ± 0.07	2.96 ± 0.33	2.91 ± 0.14
Calculated BE (MJ)	12.17	12.07	12.21	12.21	12.04	12.18	12.27

Control: no Met; LI, LII, LIII—diets with 0.3, 0.6, and 1.4 g/kg Met, respectively. Control: 1.4 g/kg Met; FI: Met-Met; FII: 50% Met + 50% Met-Met.

**Table 2:** Mean (±SD) results of carcass and shell characteristics at the end of Experiment I (Met level) and Experiment II (methionine form).

Experiment I								
Indicates	N	Control Group	Experimental Groups			SEM	*p*-Value	
			LI	LII	LIII			
Carcass weight (g)	50	6.11 ± 1.19 ^a^	5.65 ± 1.12 ^a^	5.99 ± 1.29 ^a^	6.62 ± 1.33 ^b^	0.18	0.0017	
Shell weight (g)	50	0.77 ± 0.20 ^a^	0.76 ± 0.19 ^a^	0.82 ± 0.21 ^a^	1.20 ± 0.30 ^b^	0.03	<0.0001	
Share of the carcass in total body weight (%)	50	88.87 ± 1.51 ^c^	88.20 ± 1.91 ^bc^	88.01 ^b^ ± 1.67	84.72 ± 1.83 ^a^	0.00	<0.0001	
Shell shape index	50	1.42 ± 0.09 ^c^	1.23 ± 0.12 ^b^	1.14 ± 0.11 ^a^	1.15 ± 0.06 ^a^	0.01	<0.0001	
Solidity index (g/cm^2^) × 100	50	12.22 ± 2.06 ^b^	10.63 ± 1.93 ^a^	11.01 ± 1.89 ^a^	14.13± 2.43 ^c^	0.30	<0.0001	
Crushing force of the shells (N)	20	20.43 ± 7.87 ^a^	19.35 ± 5.08 ^a^	25.40± 10.15 ^a^	48.05 ± 13.96 ^b^	2.20	<0.0001	
Mature individuals (%)	50	42.00 ± 4.99	32.00 ± 4.71	32.00 ± 4.71	54.00 ± 5.04	0.07	0.0775	
**Experiment II**								
**Indicates**	**N**	**Control Group**	**Experimental Groups**			**SEM**	***p*** **-Value**	
			**FI**		**FII**			
Carcass weight (g)	50	6.57 ± 1.29 ^a^	7.65 ± 1.17 ^b^		6.62 ± 1.39 ^a^	0.18	<0.0001	
Shell weight (g)	50	1.21 ± 0.31	1.22 ± 0.25		1.13 ± 0.36	0.04	0.2377	
Share of the carcass in total body weight (%)	50	84.53 ± 2.02 ^a^	86.20 ± 2.28 ^b^		85.66 ± 2.81 ^b^	0.00	0.0024	
Shell shape index	50	1.16 ± 0.06 ^a^	1.19 ± 0.08 ^a^		1.29 ± 0.10 ^b^	0.01	<0.0001	
Solidity index (g/cm^2^) × 100	50	13.57 ± 2.51	14.62 ± 2.65		15.02 ± 3.81	0.43	0.0524	
Crushing force of the shells (N)	20	49.73 ± 14.45	46.22 ± 18.45		42.45 ± 15.45	3.62	0.3702	
Mature individuals (%)	50	52.00 ± 5.05	66.00 ± 4.79		62.00 ± 4.90	0.07	0.3432	

No-Met Control diet, L I, L II, and L III diet with Met dose: 0.3, 0.6, and 1.4 g/kg feed, respectively. Met Control diet, FI—Met-Met, FII—50% Met and 50% Met-Met. The means indicated with different superscripts (a, b, c) are significantly different (*p* < 0.05).

**Table 3:** Mean (± SD; n = 6) proximate composition of snail carcasses and amino acid content (% of DM) in Experiment I (Met level) and Experiment II (methionine form).

Experiment I						
Item	Control Group	Experimental Groups			SEM	*p*-Value
		LI	LII	LIII		
Crude protein	64.42 ± 2.01 ^a^	71.18 ± 2.63 ^b^	69.83 ± 3.09 ^b^	63.05 ± 3.81 ^a^	1.21	0.0002
Ether extracts	2.55 ± 0.31 ^bc^	2.75± 0.16 ^c^	1.75± 0.20 ^a^	2.37 ± 0.07 ^b^	0.08	<0.0001
Crude ash	8.79 ± 0.25	9.34 ± 0.51	9.04 ± 0.52	8.65 ± 0.64	0.20	0.1144
Met (mg/g DM)	9.30 ± 1.11 ^a^	10.37 ± 0.98 ^bc^	10.00 ± 0.30 ^ab^	11.03 ± 0.61 ^c^	0.33	0.0119
Cys (mg/g DM)	7.88 ± 1.54	7.36 ± 0.34	8.09 ± 0.68	7.23 ± 0.75	0.38	0.3516
**Experiment II**						
**Item**	**Control Group**	**Experimental Groups**			**SEM**	***p*** **-Value**
		**FI**	**FII**			
Crude protein	64.22 ± 3.92	67.05 ± 2.79	64.60 ± 1.32		1.19	0.3077
Ether extracts	2.40 ± 0.07 ^b^	1.71 ± 0.20 ^a^	1.86± 0.14 ^a^		0.07	<0.0001
Crude ash	9.01 ± 0.67	8.73 ± 0.54	8.90 ± 0.38		0.22	0.5504
Met (mg/g DM)	11.05 ± 0.59 ^b^	11.92 ± 0.93 ^b^	9.70 ± 0.76 ^a^		0.32	0.0007
Cys (mg/g DM)	7.24 ± 0.71	7.49 ± 0.59	7.77 ± 0.77		0.29	0.4370

No-Met Control diet, L I, L II, and L III diet with Met dose: 0.3, 0.6, and 1.4 g/kg feed, respectively. Met Control diet, FI—Met-Met, FII—50% Met and 50% Met-Met. The means indicated with different superscripts (a, b, c) are significantly different (*p* < 0.05).

**Table 4:** The mean (± SD; n = 9) mineral content of snail carcasses (in DM) and shells (in FM) in Experiment I (Met level) and Experiment II (methionine form).

Experiment I							
Item		Control Group	Experimental Groups			SEM	*p*-Value
			LI	LII	LIII		
**Ca (%)**	Carcass	1.16 ± 0.05 ^a^	1.64 ± 0.04 ^c^	1.48 ± 0.01 ^b^	1.49 ± 0.11 ^b^	0.03	<0.0001
	Shell	34.71 ± 2.11 ^a^	36.07 ± 0.51 ^ab^	36.98 ± 1.44 ^b^	38.74 ± 1.38 ^c^	0.49	<0.0001
**Fe (mg/kg)**	Carcass	110.90 ± 10.45 ^d^	97.66 ± 4.53 ^c^	84.03 ± 5.10 ^b^	68.39 ± 3.79 ^a^	2.17	<0.0001
	Shell	41.05 ± 5.72 ^c^	20.41 ± 3.65 ^b^	16.75 ± 8.50 ^ab^	13.766 ± 3.58 ^a^	1.916	<0.0001
**Na (mg/kg)**	Carcass	8142.35 ± 175.21 ^d^	7806.86 ± 167.54 ^c^	6698.48 ± 173.70 ^b^	6454.36 ± 338.10 ^a^	75.14	<0.0001
	Shell	749.65 ± 51.02 ^a^	803.36 ± 29.38 ^c^	763.04 ± 28.59 ^ab^	792.36 ± 22.75 ^bc^	11.55	0.0080
**P (mg/kg)**	Carcass	10,942.09 ± 621.02 ^a^	14,235.50 ± 886.21 ^b^	13,622.55 ± 1278.67 ^b^	11,287.65 ± 422.78 ^a^	287.94	<0.0001
	Shell	362.79 ± 24.36 ^a^	655.30 ± 79.13 ^c^	415.31 ± 21.63 ^b^	420.74 ± 55.15 ^b^	16.97	<0.0001
**Zn (mg/kg)**	Carcass	71.32 ± 2.62 ^a^	88.52 ± 6.15 ^c^	82.43 ± 4.96 ^b^	68.64 ± 3.23 ^a^	1.49	<0.0001
	Shell	2.60 ± 0.49 ^a^	4.57 ± 0.08 ^b^	3.84 ± 0.90 ^b^	4.86 ± 2.02 ^b^	0.38	0.0009
**Cu (mg/kg)**	Carcass	63.64 ± 4.56 ^d^	60.56 ± 2.47 ^c^	56.96 ± 2.38 ^b^	51.77 ± 1.20 ^a^	0.97	<0.0001
	Shell	11.05 ± 0.55 ^b^	8.30 ± 1.58 ^a^	8.54 ± 1.49 ^a^	8.55 ± 2.52 ^a^	0.56	0.0042
**Cr (mg/kg)**	Carcass	ND	0.33 ± 0.23	ND	ND	-	-
	Shell	ND	ND	ND	ND	-	-
**Co (mg/kg)**	Carcass	0.11 ± 0.01	ND	ND	ND	-	-
	Shell	ND	ND	ND	ND	-	-
**Cd (mg/kg)**	Carcass	0.96 ± 0.03 ^c^	0.59 ± 0.015 ^b^	0.52 ± 0.05 ^a^	0.57 ± 0.06 ^b^	0.01	<0.0001
	Shell	ND	ND	ND	ND	-	-
**Pb (mg/kg)**	Carcass	ND	ND	ND	ND	-	-
	Shell	ND	ND	ND	ND	-	-
**Experiment II**							
**Item**		**Control Group**	**Experimental Groups**			**SEM**	***p*** **-Value**
			**FI**	**FII**			
**Ca (%)**	Carcass	1.50 ± 0.1	1.39 ± 0.18	1.45 ± 0.19		0.06	0.4066
	Shell	38.74 ± 1.38 ^b^	36.11 ± 1.97 ^a^	36.95 ± 0.56 ^a^		0.48	0.0022
**Fe (mg/kg)**	Carcass	68.19 ± 3.72	69.37 ± 10.06	65.39 ± 5.72		2.34	0.4686
	Shell	13.76 ± 3.58 ^a^	12.13 ± 1.81 ^a^	23.18 ± 4.39 ^b^		1.14	<0.0001
**Na (mg/kg)**	Carcass	6452.32 ± 338.10	6362.59 ± 764.05	5917.78 ± 471.08		184.59	0.1106
	Shell	792.36 ± 22.75 ^a^	806.55 ± 18.50 ^a^	946.93 ± 31.67 ^b^		8.31	<0.0001
**P (mg/kg)**	Carcass	11,237.61 ± 422.78	11,738.28 ± 653.38	11,121.29 ± 543.66		182.70	0.0659
	Shell	420.74 ± 55.15	456.50 ± 9.20	423.96 ± 12.01		11.01	0.0572
**Zn (mg/kg)**	Carcass	68.34 ± 3.23	72.17 ± 3.98	69.40 ± 3.43		1.19	0.1068
	Shell	4.86 ± 2.02	5.39 ± 1.05	4.28 ± 1.13		0.49	0.2918
**Cu (mg/kg)**	Carcass	51.97 ± 1.20 ^a^	61.87 ± 4.30 ^c^	57.46 ± 4.80 ^b^		1.26	<0.0001
	Shell	8.55 ± 2.52 ^b^	8.72 ± 0.85 ^b^	6.77 ± 0.65 ^a^		0.53	0.0268
**Cr (mg/kg)**	Carcass	ND	ND	ND		-	-
	Shell	0.16 ± 0.22 ^a^	ND	0.91 ± 0.69 ^b^		0.17	0.0172
**Co (mg/kg)**	Carcass	ND	ND	ND		-	-
	Shell	ND	ND	ND		-	-
**Cd (mg/kg)**	Carcass	0.58 ± 0.06	0.61 ± 0.03	0.57 ± 0.02		0.01	0.0717
	Shell	ND	ND	ND		-	-
**Pb (mg/kg)**	Carcass	ND	ND	ND		-	-
	Shell	ND	ND	ND		-	-

No-Met Control diet, L I, L II, and L III diet with Met dose, respectively: 0.3, 0.6, 1.4 g/kg feed. Met Control diet, FI—Met-Met, FII—50% Met and 50% Met-Met. ND—not detected. The means indicated with different superscripts (a, b, c) are significantly different (*p* < 0.05).

## Data Availability

The raw data supporting the conclusions of this article will be made available by the authors on request.

## Abbreviations

The following abbreviations are used in this manuscript:

Met-Met	Conjugated form of methionine
Met	Methionine
DM	Dry matter
BW	Body weight
SW	Shell width
DPPH	2,2-difenylo-1-pikrylohydrazyl
TAC	Total Antioxidant Capacity
TBARS	Thiobarbituric acid reactive substances
SAM	S-adenosylmethionine
GSI	gonadal specific index
DNA	deoxyribonucleic acid
RNA	ribonucleic acid
INRA	French National Institute for Agricultural Research
CG	Control Group
AOAC	Association of Official Analytical Chemists
MDA	malondialdehyde
TEP	1,1,3,3-tetraethoxypropane
BHA	butyl hydroxyanisole
TBA	thiobarbituric acid
DTNB	5,5′-dithiobis (2-nitrobenzoic acid
TCA	trichloroacetic acid
GSH	glutathione
HCl	hydrochloric acid
Cys	cysteine
ALF1	anti-lipopolysaccharide factor 1
proPO	Crustin-1, prophenoloxidase
CncC	cap’ n’ collar isoform C
ADA	adenosine deaminase
GPT	glutamate transaminase
GOT	aspartate aminotrafserase
CFH	total antioxidative capacity in cell-free hemolymph
ACP	acyl carrier protein
LZM	lysozyme
TOR	Target of Rapamycin
